# Comparison of Postoperative Pulmonary Complication Indices in Elective Abdominal Surgery Patients

**Published:** 2020-01

**Authors:** Askin Gülsen, Oguz Kilinc, Kemal Can Tertemiz, Tuncay Ekice, Türkan Günay

**Affiliations:** 1 Department of Pneumology, University of Lübeck, Germany,; 2 Department of Pneumology, Dokuz Eylül University School of Medicine, Izmir, Turkey,; 3 Department of General Surgery, Dokuz Eylül University School of Medicine, Izmir, Turkey,; 4 Department of Public Health, Dokuz Eylül University School of Medicine, Izmir, Turkey

**Keywords:** Preoperative assessment, Risk indexes, Elective abdominal surgery, Postoperative pulmonary complication

## Abstract

**Background:**

Postoperative pulmonary complications (PPC) are important problems that prolong hospital stays by increasing morbidity and mortality of patients. Early identification of risky cases through preoperative evaluation is important for reducing the complications that may be seen in patients postoperatively. The aim of this study is to calculate, evaluate and compare the risk indices for PPC in patients who will undergo elective abdominal surgery.

**Materials and Methods:**

One hundred twenty-four patients who were hospitalized for elective abdominal surgery were included in this prospective observational study. American Society of Anesthesiologists (ASA), Epstein and Shapiro scores, respiratory failure index (RFI), pneumonia risk indexes (PI) and scores were calculated preoperatively. Patients were re-evaluated at the 48th postoperative hour, and one-week follow-up was performed. The patients with PPCs are recorded.

**Results:**

The mean PPC rate was 36.8%. Based on this, pleural effusion was observed in 18.5%, prolonged mechanical ventilation in 8.9%, atelectasis in 9.7%, and respiratory failure in 5.7%, bronchospasm in 4.0%, and pneumonia in 3.2% of patients. An increased risk in PPC was determined if ASA were above 3 (odds ratio, [OR], 7.06; <0.001), PI scores were above 3 (OR, 6.67; <0.001), RFI score were above 4 (OR, 6.30, p:0.001) and Shapiro score above 2 (OR, 20.01; <0.001), respectively.

**Conclusion:**

The Shapiro index is the strongest predictor of pulmonary complications, whereas the PI is the strongest predictor of morbidity risk. However, RFI and the PI are equally valuable for predicting respiratory complications and may prove to be useful in abdominal surgeries for preoperative assessment.

## INTRODUCTION

Postoperative pulmonary complications (PPCs) in abdominal surgery are important factors that can prolong the length of hospital stays, as well as be a cause of morbidity and mortality (1). The preoperative assessment (PA) plays an important role in predicting complications and taking precautions, it is often performed to collect history and the results of a physical examination, chest X-ray, respiratory function tests (RFT), as well as arterial blood gas (ABG) and exercise tests, as required (2).

**Figure 1.**
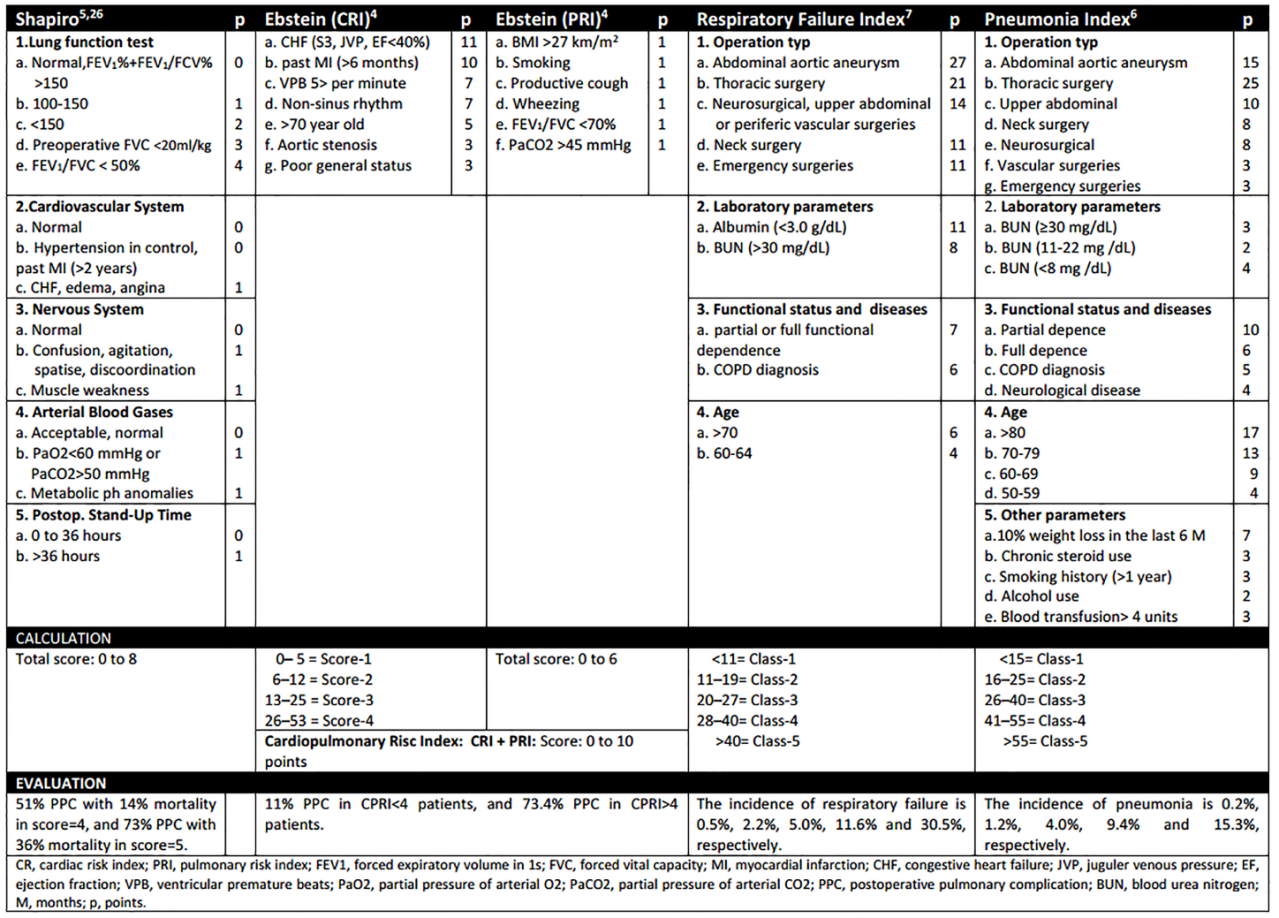
Calculation and comparison of all preoperative indexes

Various risk indices have been developed for preoperative evaluation in recent years. These include the American Society of Anesthesiologists’ (ASA) physical status classification, Epstein’s cardiopulmonary risk index (CPRI), the Shapiro index, the pneumonia risk index, and the respiratory failure index (RFI) (3–7) (Figure-1). These indices predict postoperative prognosis and complications.

The ASA classification identifies the general physical state of patients and is commonly used for preoperative assessment in all hospitals. Patients with ASA classification II and above are known to have increased PPCs and other complications. The incidence of mortality in the ASA score is reported to be 0.1% in ASA-I, 0.7% in ASA-II, 3.5% in ASA-III, 18.3% in ASA-IV, and 93.3% in ASA-V(3).

Epstein et al. developed the CPRI in 1993 by modifying Goldman’s cardiac risk index. This index includes the cardiac risk index (CRI) and pulmonary risk index (PRI). The CRI has been used for a long time to detect perioperative cardiac and pulmonary complications. The total score is 10 in the CPRI assessment. The postoperative cardiopulmonary complication rate has been found to be 73.4% in patients with a score greater than 4 and 11% in those with a score less than 4 (4).

The Shapiro index was developed after the ASA and the CPRI and includes parameters defining spirometry, blood gas, cardiovascular system, nervous system, and postoperative recovery period. It can appear to resemble the CPRI but contains different nervous system and muscle strength examinations (5).

Arozullah et al. developed RFI and the pneumonia risk index in 2000 and 2001 with multicenter studies. These two indices are basically similar to one another; but together, they evaluate a large number of parameters. Additionally, the pneumonia risk index contains several more parameters. Using both indices, the patients were distributed among 5 risk groups, and the complications were recorded (6,7).

There are a limited number of studies in the literature comparing these indices with PPC. Hence, we planned to investigate the value of these indices and the reasons for an increase in the risk of postoperative complications in patients who are scheduled for an elective abdominal surgery.

## MATERIALS AND METHODS

One hundred twenty-four patients who were hospitalized for elective abdominal surgery in the Dokuz Eylül University Department of General Surgery were included in this prospective observational study. The inclusion criteria were as follows: (i) elective abdominal surgery; (ii) patients over 18 years of age; (iii) all types of incisions; (iv) smoking cessation at least 48 hours prior to surgery. Exclusion criteria were as follows: (i) patients who underwent emergency operations; (ii) inguinal hernia operations; (iii) organ transplantations; (iv) patients who were re-operated upon within 90 days; (iv) patients under regional or spinal anesthesia; (v) patients age under 18 years. Ethical committee approval was obtained from the Dokuz Eylül University Ethics Committee. Informed consent form was obtained from all patients.

### Preoperative and postoperative assessment

The patients were assessed within the last three days before surgery. Demographic data of all patients were recorded. All patients underwent pulmonary function test with the Sensormedics-Vmax 22 spirometer and postero-anterior chest X-rays with a digital imaging system. Arterial blood gases of patients with FEV_1_ < 50% were examined using a Nova Biomedical Stat Profile M/2/3 device at a central laboratory in Dokuz Eylul University Hospital. All risk indices (ASA, CPRI, RFI and pneumonia risk index, Shapiro) were calculated by the same pneumologist.

Patients were re-evaluated at the 48^th^ postoperative hour, and a one-week follow-up was performed. The type of operation, its duration, the incision size and shape, and whether or not the operation was performed by laparoscopy were recorded. Extubation time and complications were determined for patients who were in the intensive care unit for more than 48 hours. Respiratory symptoms and physical conditions for all patients were checked. Chest X-rays and arterial blood gas analyses were performed in cases in which the patient showed symptoms or there were pathological examination findings. All postoperative complications (atelectasis, bronchospasm, re-intubation, prolonged mechanical ventilation, pleural effusion, respiratory failure, COPD exacerbation, pneumonia, and acute respiratory distress syndrome) were recorded. Definitions were obtained from the “European Perioperative Clinical Outcome Definition” (8). All patients were checked at the end of 1 month, and the survival rate was calculated.

### Statistical analysis

The data obtained by the surveys were uploaded to the SPSS for Windows 14.0 statistical program (SPSS, Chicago, Illinois, USA), and statistical analyses were performed. During the analysis, the capability to determine the frequency of complications in the PA indices was assessed by determining the odds ratios and the area beneath the chi-squared curve test. The confidence interval was calculated using the Miettinen formula. The rates of postoperative respiratory complications in abdominal surgeries were compared to patient and surgery parameters. Factors affecting PPC were assessed using a chi-squared (χ^2^) analysis, the differences between grouped data were assessed using a student-t test, and the parameters that did not fulfill parametric conditions (determined by measurement) were assessed using the Mann Whitney-U test. The capability to determine the frequency of complications of the indices that determine postoperative complications was calculated using the chi-squared (χ^2^) analysis beneath the curve and by measuring the odds ratios (OR) and the 95% confidence interval, which was calculated using the Miettinen formula. All results were evaluated with a 95% confidence interval and a significance level of p<0.05.

## RESULTS

One hundred and twenty-four patients scheduled to undergo elective abdominal surgery were included in the study. Demographic data of patients, distribution of preoperative risk indices, clinical characteristics of patients are shown in Table 1. However, a statistically significant relationship was found between the risk of developing PPC and age, preoperative pathological respiratory examination findings, and postoperative intensive care need. We found at least one case of PPC in 45 (36.2%) of the patients in all abdominal operations, and non-respiratory complications were detected in 21 (16.9%) of the patients (Table 2). In the following 1 month, 9 (7.2%) patients lost their lives. PPCs were seen in 48.2% of upper abdominal operations (27/56), 37.5% of lower abdominal operations (15/40), and 10.7% of laparoscopic operations (3/28) (Table 3). During the follow-up period, the rate of severe PPC was found to be 19.6% in upper abdominal operations (11/56), 20.0% in lower abdominal operations (8/40) and 0% in laparoscopic operations (0/28) according to an analysis performed by excluding remitting atelectasis and pleural effusion.

**Table 1.** Distribution of Demographic Data and Indices (n:124)

	**n**	**%**	**Index**	**n**	**%**
**Gender**			**ASA**		
Female	63	50.8	I	48	38.7
Male	61	49.2	II	56	45.2
**BMI** ≥30	23	18.5	III	19	15.3
**Smoking Status**			IV	1	0.8
Active smoker	32	25.8	**CPRI**		
Ex-smoker	38	30.6	0	33	26.6
Never smoker	40	32.3	1	56	45.2
Passive exposure	14	11.3	2	27	21.8
**History of Lung Disease**			3	3	2.4
No	111	89.5	4	4	3.2
Yes	13	10.5	5	1	0.8
**Preop.Respiratory Complaint**			**Shapiro**		
No	99	79.9	0	91	73.4
Yes	25	20.1	1	24	19.4
**Preop.Positive Examination**			2	6	4.8
No	101	81.5	3	3	2.4
Yes	23	18.5	**Resp. Failure Index**		
**Surgery Localization**			I	52	41.9
Upper	56	45,2	II	45	36.3
Under	40	32.3	III	15	14.5
Laparoscopic	28	22.6	IV	8	6.5
			V	1	0.8
**Incision Types**			**Pneumonia Risk Index**		
Vertical	59	47.6	I	48	38.7
Horizontal	11	8.8	II	43	34.7
Subcostal	26	20.9	III	29	23.4
Laparoscopic	28	22.5	IV	4	3.2

**Table 2.** Postoperative Complications in Abdominal Operations

**Pulmonary Complications**	**n**	**%**	**Non-Pulmonary Complications**	**n**	**%**
Pleural effusion	23	18.5	Infection	8	6.4
Atelectasis	12	9.7	Sepsis	3	2.4
Prolonged mechanical ventilation	11	8.9	Wound infection	3	2.4
Respiratory Failure	7	5.6	Peritonitis	1	0.8
Bronchospasm	5	4.0	Perihepatic abscess	1	0.8
Pneumonia	4	3.2	Atrial Fibrillation	6	4.8
ARDS	2	1.6	Volume load and Cardiac failure	2	1.6
Reintubation	1	0.8	Bleeding	2	1.6
			Acute Renal Failure	2	1.6
			Hepatic Encephalopathy	1	0.8
			Acute arterial occlusion	1	0.8

There are multiple complications in the same patient.

**Table 3.** Postoperative pulmonary and non-pulmonary complications according to surgery localization

**Complications**	**Upper Abd. n (%)**	**Under Abd. n (%)**	**Laparoscopic n (%)**
**PPC (at least 1)**	27 (48.2)	15 (37.5)	3 (10.7)
Atelectasis	4 (7.1)	5 (12.5)	3 (10.7)
Pleural effusion	17 (30.3)	6 (15.0)	0 (0)
Serious PPC	11 (19.6)	8 (20.0)	0 (0)
**Non-Pulmonary Complications**	11 (19.6)	10 (25.0)	0 (0)

Abd: Abdominal, there are multiple complications in the same patient.

The durations of the operations were 258.1 ± 101.4 min for upper abdominal surgeries, 242.1 ± 88.6 min for lower abdominal surgeries, and 106.7 ± 38.3 min for laparoscopic surgeries. The duration of surgery was longer in patients with PPC (270.56 ± 110.11 vs. 189.30 ± 91.20 min.; p < 0.001). In addition, it was observed that the frequency of pulmonary complications increased as the duration of intensive care stay increased after surgery (p < 0.001). Respiratory and non-respiratory complications were observed in all patients requiring prolonged postoperative mechanical ventilation. Therefore, the 1-month mortality of these patients was recorded as 54.5%.

When the PPCs were compared by incision types, there were no statistically significant differences between vertical, horizontal, and subcostal incisions (p>0.05); increase in incision size (p=0.02) and duration of operation (p < 0.001) statistically increased PPC risk. As the fall rate of albumin values in the postoperative period grew higher than that of the preoperative period, the risk of developing PPC also increased (p=0.002).

When the respiratory function tests were compared to the PPC, there was a statistically significant relationship between FEV1, FVC, FEV1 / FVC, FEF_25–75_, and percentages and the development of PPC (Table 4).

**Table 4.** Correlation of various risk factor with PPC

**Parameters**	**n:**	**PPC (-)**	**PPC (+)**	**P** **-value**
Female, n (%)	63	45 –(71.4)	18 –(28.6)	0.069
Male, n (%)	61	34 –(55.7)	27 –(44.3)	
Age, year	124	53.32 ± 15.0	62.69 ± 14.7	0.001
BMI, kg/m^2^	124	26.13 ± 4,98	25.72 ± 4.86	0.66
**Smoking duration (pack/year)**	124	14.82 ± 22.3	20.57 ± 24.0	0.18
0–19.99, n (%)	21	16 – (76.1)	5 – (23.8)	0.01
> 20.00, n (%)	49	26 – (53.1)	23 – (46.9)	<0.001
**Smoking status**				
Never, n (%)	54	54 – (100)	0 – (0)	0.01
Ex-smoker, n (%)	38	37 – (97.4)	1 – (2.6)	
Active smoker, n (%)	32	28 – (87.5)	4 – (12.5)*	
**Preop.Positive Examination findings**				
Negative, n (%)	101	69 – (68.3)	32 – (31.7)	0.03
Positive, n (%)	23	10 – (43.5)	13 – (56.5)*	
**Incision Types**				
Vertical, n (%)	59	35 – (59.3)	24 – (40.7)	0.002
Horizontal, n (%)	11	4 – (36.3)	7 – (63.7)	
Subcostal, n (%)	26	15 – (57.7)	11 – (42.3)	
Laparoscopic, n (%)	28	25 – (89.3)	3 – (10.7)*	
Size of the incision, cm	96	10.93 ± 13.77	22.07 ± 16.54	0.02
Duration of surgery, min	124	189.30 ± 91.20	270.56 ± 110.11	<0.001
Albumin Gradient, mg/dl	124	−0.54 ± 0.61	−0.97 ± 0.76	0.002
Durations of stay in intensive care, hour	124	0.92 ± 4.49	37.58 ± 69.74	<0.001
**Prolonged Postope. intensive care**				
Negative, n (%)	97	76 – (78.4)	21 – (21,6)	<0.001
Positive, n (%)	27	3 – (11.1)	24 – (88,9)*	
**PFT parameters**				
FEV_1_ L		2.75 ± 0.83	2.34 ± 0.81	0.01
FEV_1_ %		94.37 ± 20.62	79.18 ± 15.0	0.001
FVC L		3.37 ± 0.95	2.89 ± 0.85	0.007
FVC %		96.76 ± 20.28	83.16 ± 13.7	0.001
FEV_1_/FVC %		79.30 ± 6.4	75.30 ± 8.8	0.005
FEF_25–75_		2.77 ± 1.16	2.10 ± 0.93	0.001
FEF_25–75_%		82.40 ± 25.8	69.60 ± 24.2	0.005

Data are shown as mean ± standard deviation; PPC, postoperative pulmonary complication; BMI, body mass index; PFT, pulmonary function test; Postope, postoperative; Preop, preoperative

### Risk indices and respiratory complications

When the CRI and PRI were evaluated in terms of respiratory complications, the odds ratio was found to be 2.34 (0.16<OR < 4.51) and 3.15 (0.40<OR < 5.89). However, when this index was used with the CPRI, it was observed that a score of 3 and above showed the complications to be much better. When the CPRI was 3 and above, the odds ratio was 9.33 (3.72 < OR < 14.92). The odds ratio was 7.06 (3.10 < OR < 11.01) when ASA was III and above; it was 6.30 (1.76 < OR < 7.56) when the RFI was 4; and it was 6.67 (3.33 < OR < 9.45) when the pneumonia risk index was>3. The odds ratio was found to be 20.01 (9.30<OR < 30.41) on the Shapiro index of 3 and above, making it the best predictor of respiratory complications (Table 5). The PPC distribution of all patients according to risk indices is presented in Table 6.

**Table 5.** Comparison of indices with PPC

**Indices with PPC**	**OR**	**%95 CI**	**p-value**
Epstein index (PRI) ≥ 2	2.34	0.16< OR <4.51	0.06
Epstein index (CRI) ≥ 2	3.15	0.40< OR <5.89	0.02
RFI ≥ 4	6.30	1.76< OR <7.56	0.001
Pneumonia risk index ≥ 3	6.67	3.33< OR <9.45	<0.001
ASA ≥ III	7.06	3.10< OR <11.01	<0.001
Epstein index (CPRI) ≥3	9.33	3.72< OR <14.92	<0.001
Shapiro index ≥ 3	20.01	9.30< OR <30.41	<0.001

PPC, postoperative pulmonary complication; RFI, respiratory failure index; PRI, pulmonary risk index; ASA, American Society of Anesthesiologists physical status classification; CPRI, cardiopulmonary risk index; OR; odds ratio; CI, confidence interval.

**Table 6.** Distribution of PPC according to indices (n:45)

**Index**	**PPC (−) n (%)**	**PPC (+) n (%)**	**p-value**	**Index**	**PPC (−) n (%)**	**PPC (+) n (%)**	**p-value**
**ASA**			<0.001	**Epstein**			<0.05
Stage-1	38 – (79.2)	10 – (20.8)		Risk-0	28 – (84.8)	5 – (15.2)	
Stage-2	34 – (60.7)	22 – (39.3)		Risk-1	34 – (60.7)	22 – (39.3)	
Stage-3	7 – (36.8)	12 – (63.2)		Risk-2	14 – (51.9)	4 – (48.1)*	
Stage-4	0	1 – (100)		Risk-3	2 – (66.7)	2 – (33.3)	
Stage-51	0	0		Risk-4	1 – (20.0)	4 – (80.0)	
**RFI**			<0.001				<0.001
Risk-1	42 – (80.8)	10 – (19.2)		**Shapiro**			
Risk-2	25 – (55.6)	20 – (44.4)		Risk-0	65 – (71.4)	26 – (28.6)	
Risk-3	9 – (50.0)	9 – (50.0)*		Risk-1	13 – (54.2)	11 – (45.8)	
Risk-4	3 – (37.5)	5 – (62.5)		Risk-2	1 – (16.7)	5 – (83.3)*	
Risk-5	0	1 – (100)		Risk-3	0	3 – (100)	
**Pneumonia RI**			<0.001				
Risk-1	39 – (81.3)	9 – (18.8)					
Risk-2	27 – (62.8)	16 – (37.2)					
Risk-3	13 – (44.8)	16 – (55.2)*					
Risk-4	0	4 – (100)					

* p value was calculated between this group and other groups.

Groups with 5 or less patients were included in the previous group during statistical analysis.

PPC, postoperative pulmonary complication; RFI, respiratory failure index; RI, risk index; ASA, American Society of Anesthesiologists physical status classification;

### Indices and death

At the end of the first postoperative month, 9 of 124 patients (7.2%) died. The mean duration of operation was 298.33 ± 99.40 min (duration of operation range 145–480 min). Eight of the patients required postoperative intensive care, while 1 patient began to be monitored during postoperative recovery. The mean duration of ICU follow-up was 67.11 ± 51.02 hours. Respiratory and non-respiratory complications were present in all patients. Four patients had multiple respiratory complications. Prolonged mechanical ventilation was seen in 6 patients, pleural effusion in 5 patients, respiratory failure in 3 patients, ARDS in 2 patients, reintubation in 1 patient, and atelectasis in 1 patient. When the relationship between the patients who died and the indices was examined, it was seen that the ASA index was not well distributed, and there was no difference among the groups. Despite RFI and the pneumonia risk index being complication-specific indices, mortality was higher in patients with a risk index of 3 and above. The pneumonia risk index had a meaningful mortality distribution and was the most meaningful in statistical analysis (p<0.001).

## DISCUSSION

The main findings of the study are that the indices are useful in predicting PPCs. Although RFI, pneumonia risk index, and ASA are close to each other for prediction purposes, the Shapiro index differs from others with its stronger prediction of PPCs.

In general, PPCs are seen in 5–10% of major abdominal surgeries (7,9). Therefore, pulmonary complications are more common following upper abdomen, thoracic and abdominal aortic aneurysm repair surgeries (10). The incidence of atelectasis in abdominal surgeries is reported to be 20–69%, while postoperative pneumonia is reported to be 9–40% (11). However, it is necessary to perform a preoperative assessment on patients before they undergo surgery and to take necessary precautions to alleviate any potential complications. PPCs are important problems that prolong hospital stays by increasing morbidity and mortality of patients (12).

In our study, similar to the literature, PPC was 36.8%, pleural effusion 18.5%, atelectasis 9.7%, and pneumonia 3.2%. There are also studies suggesting that complications occur more frequently during upper abdominal surgeries than during lower abdominal surgeries (13–33% vs. 0–16%) (13). When examined in terms of operation localization, at least 1 PPC (mild or severe) was found in 48.2% of upper abdominal surgeries, 38.5% of lower abdominal surgeries, and 10.7% of laparoscopic surgeries. These ratios, which can be considered high in comparison to the literature, are thought to be due to mild complications (effusion, bronchospasm, atelectasis), which we included within the term PPC in our study. When these complications are excluded, the severity of PPC is 11/56, or 19.6%, in upper abdominal surgeries and 8/40, or 20.0%, in lower abdominal surgeries. The most common complication detected in upper abdominal surgeries in our study was pleural effusion (17/56, 30.3%).

Age is an important factor in the development of PPC. In a relevant study by Djokovic et al., postoperative 30-day mortality was found in 6.2% of patients over 80, and less than 1% of these patients were in the ASA II class (14). Similarly, we have found that the frequency of complications increases with age.

In a meta-analysis conducted in 2014, PPC was reported 2.0% in laparoscopic cholecystectomy and 5.8% in open cholecystectomy (15). In our study, a PPC rate of 10.7% was found in laparoscopic cholecystectomy. This ratio, which is higher than that found in the literature, is thought to be due to the fact that the general overall condition of patients scheduled for cholecystectomy operation is better, their complaints are fewer than other patients, and, accordingly, they do not tend to quit smoking early on. Active smokers made up 39.3% of the patients who were to undergo laparoscopic cholecystectomy. Bronchospasm was seen in 12.5% of patients who stopped smoking in the last 48 hours. In addition, PPC was seen in 46.9% of patients with a history of smoking 20 or more packets per year. Smetana et al. reported that a certain period of learning was required to make patients quit smoking during the preoperative period and that complications occurred in 57% of patients who did not quit, as opposed to those 33% of patients who quit 8 weeks prior to surgery (16). The results obtained here can be used in another study to further investigate preoperative smoking cessation rates according to disease symptoms and to determine how many patients who quit smoking in the pre-operative period continued not to smoke post-operatively.

Although there are contradictory studies regarding the type and size of the incision, it is reported that complications are more common with a vertical incision (1). In a study conducted by Kumar et al. in 2018, no difference was found between the type and size of incisions in abdominal surgeries (17). In our study, no difference was found between the incisions when all the abdominal surgeries were considered, but there were more complications in these three types of incisions than in the laparoscopy. The incision sizes of patients with PPC were found to be longer (average 22.07 ± 16.54 cm vs. average 10.93±13.77 cm, p=0.02).

Another important point for PPCs is duration of anesthesia and surgery. PPCs had been reported more frequently in surgeries lasting more than 4 hours (9, 12,18). Similarly, in our study, the duration of surgery in cases with PPC were longer than 4 hours (270.56 ± 110.11 min).

Prolonged anesthesia duration causes atelectasis in the lung. In recent years, lung protective low tidal volume ventilation (6–8 ml/kg), and positive-end expiratory pressure (PEEP; 6–8 cm H_2_O) are preferred to reduce this issue (19). In another study, it has been reported that PPC risk increases with increasing duration of surgery (20). In this study, OR was reported 4.9 (2.4–10.1) and 9.7 (4.7–19.9) in operations over 2 hr and 3 hr, respectively.

Literature reviews reveal that there are many studies reporting more complications in patients who stay in the intensive care unit and prolonged ventilator support over 48 hours (21). In our study, when the patients’ durations of stay in intensive care unit were examined, 88.9% of the patients who had postoperative intensive care had PPCs, with more complications seen in those who stayed for an average of 37.5 hours. Başoğlu et al. (22) reported PPCs in 42.9% of patients who spent more than 48 hours in intensive care following elective upper abdominal surgery, while Hofer et al. (21) reported that 30–50% of patients experienced them, as well as a 10–60% rate of mortality, depending on the severity of respiratory insufficiency in patients.

It is known that if FEV_1_ or FEV_1_/FVC is below 70% of the expected value, postoperative complications increase, but there is not always a result consistent with these values. There are studies reporting that (23, 24) pulmonary complications develop more frequently postoperatively in patients with a FEV1 value of less than 1.25 L as opposed to studies reporting that even when the FEV_1_ is very low, mortality and morbidity levels do not increase (25,26). In our study, pulmonary complications were more frequent in patients with a FEV_1_ of 70% less than predicted (p=0.03).

ASA scoring is a practically computable scoring system that has been used for many years, predicting postoperative mortality and respiratory complications in research (3). Shapiro et al. reported 75% pulmonary complications and 18% mortality in patients with ASA levels of IV and above (5). In our study, respiratory complications were more frequent in patients with ASA levels of III and above (OR, 7.06; <0.001). ASA scoring was also better at predicting non-respiratory complications, and patients with a score of III and above were found to have more non-respiratory complications (OR, 5.92; <0.001).

In patients with a CPRI score of 4 points or higher, the rate of postoperative cardiopulmonary complications was found to be 73.4%, and it was found to be 11% in those with lower scores (4). This index has been examined both separate from and within in our research. When separated as CRI and PRI, no statistically significant difference was found between respiratory complications of patients with a score of 0 and above and 2. However, when this index was considered to be CPRI, pulmonary complications were more frequent (OR, 9.33; <0.001) in patients with a total score of 3 or more. Regarding non-respiratory complications, PRI alone did not have statistical significance (p=0.58), but it was found that PRI helped predict non-respiratory complications when CRI score was 2 and above (OR, 23.75; <0.001). In our study, it was also found that cardiac index was important in showing non-respiratory complications alone, but this index should be used as a cardiopulmonary risk index.

Arozullah et al. found that respiratory insufficiency was found in 5% of patients with RFI-3, 11.6% of patients with RFI-4, and 30.5% of patients with RFI-5 (7). Concerning the pneumonia risk index, 4% of patients with a score of 3, 9.4% of patients with a score of 4, and 15.3% of patients with a score of 5 had pneumonia (6). In our study, PPCs were found to be more frequent in patients with RFI score of 4 and above (OR, 6.30; <0.001) and a pneumonia risk index score of 3 and above (OR, 6.60; <0.001). Because these two indices have similar evaluation parameters and results, it has been found that they are equally accurate for predicting PPC.

When the relationship between the patients who died and the indices were examined, the evaluation was limited due to the fewer operations performed on patients in the high-risk group and the low number of patients in the groups. In the statistical analysis of combined groups, the pneumonia risk index was the most significant index in terms of indicating mortality risk.

In a study on the Shapiro index, Wong et al. reported pulmonary complications in 51% of patients when the index was 4 points or above, and in 73% of patients when the same index was 5 points and above, as well as an observed mortality rate of 36% (27). This index played an important role in predicting the development of PPCs (OR, 20.01;<0.001) when the score is 2 points or above.

There are several limitation factors in our study. First, the data were obtained only from one center. Secondly, all types of abdominal surgery were evaluated together due to the small number of cases. Thirdly, cases of elective malignancy surgeries were included in the study but no distinction was made according to their stages. A multicenter study with more cases and subgroup analysis can provide more detailed information. However, our study is the first study comparing all indexes with each other.

According to the results of our study, about 1/3 of the patients continue to smoke in the preoperative period, and this lead to an increase in complications in the operations to be performed for diseases with fewer preoperative disease symptoms, such as cholelithiasis. It is strongly recommended that patients in the preoperative period have increased awareness and that smoking cessation treatments be made more effective. Simple respiratory function tests performed on patients with risk factors have also been helpful in predicting complications. In addition, patients over 62 years of age with a history of smoking more than 20 packets per year, a preoperative smoking habit, and a scheduled upper abdominal surgery, and who have a preoperative FEV_1_ and/or FEV_1_ / FVC value of less than 70%, and basically who are above ASA level III have a greater risk of developing PPCs.

## CONCLUSION

This study includes a simultaneous and comprehensive examination of the indices developed to predict PPCs. In conclusion, the Shapiro index is the strongest predictor of pulmonary complications, whereas the pneumonia risk index is the strongest predictor of morbidity risk. In addition, RFI and the pneumonia risk index are equally valuable for predicting respiratory complications and may prove to be useful in abdominal surgeries for PA.
